# Tetracycline Reduces Kidney Damage Induced by *Loxosceles* Spider Venom

**DOI:** 10.3390/toxins9030090

**Published:** 2017-03-02

**Authors:** Cinthya Kimori Okamoto, Carmen W. van den Berg, Mizuno Masashi, Rute M. Gonçalves-de-Andrade, Denise V. Tambourgi

**Affiliations:** 1 Immunochemistry Laboratory, Butantan Institute, São Paulo 05503-900, Brazil; cinthya.okamoto@butantan.gov.br (C.K.O.); rutemga2@gmail.com (R.M.G.-d.-A.); 2 Institute of Molecular and Experimental Medicine, School of Medicine, Cardiff University, Cardiff CF144XN, UK; vandenbergcw@cardiff.ac.uk; 3 Renal Replacement Therapy, Nagoya University Graduate School of Medicine, Nagoya 466-8550, Japan; mmizu@med.nagoya-u.ac.jp

**Keywords:** *Loxosceles* venom, sphingomyelinase D, kidney cells, matrix metalloproteinases (MMPs), kidney injury, tetracycline

## Abstract

Envenomation by *Loxosceles* spider can result in two clinical manifestations: cutaneous and systemic loxoscelism, the latter of which includes renal failure. Although incidence of renal failure is low, it is the main cause of death, occurring mainly in children. The sphingomyelinase D (SMase D) is the main component in *Loxosceles* spider venom responsible for local and systemic manifestations. This study aimed to investigate the toxicity of *L. intermedia* venom and SMase D on kidney cells, using both In vitro and in vivo models, and the possible involvement of endogenous metalloproteinases (MMP). Results demonstrated that venom and SMase D are able to cause death of human kidney cells by apoptosis, concomitant with activation and secretion of extracellular matrix metalloproteases, MMP-2 and MMP-9. Furthermore, cell death and MMP synthesis and secretion can be prevented by tetracycline. In a mouse model of systemic loxoscelism, *Loxosceles* venom-induced kidney failure was observed, which was abrogated by administration of tetracycline. These results indicate that MMPs may play an important role in *Loxosceles* venom-induced kidney injury and that tetracycline administration may be useful in the treatment of human systemic loxoscelism.

## 1. Introduction

Envenomation by *Loxosceles* spider is considered a serious public health problem in Brazil, with more than 7000 cases being reported annually [1]. Loxoscelism can be presented as two clinical forms: cutaneous and systemic. Local pain, fever, erythema and edema are characteristic manifestations of cutaneous loxoscelism, which may lead to ischemia evolving to necrosis [2,3,4]. Systemic loxoscelism is less common, with the highest incidence in children [2]. Symptoms can include fever, thrombocytopenia, intravascular hemolysis, platelet aggregation, persistent inflammation, and in severe cases, kidney failure and death [2,5].

Various studies have identified several of the components present in *Loxosceles* venom that may contribute to the pathology, including lipases, hyaluronidase, collagenase, sphingomyelinases and phosphatases [2,6]. In a series of studies, we have purified, characterized, cloned and expressed sphingomyelinases D (SMase D) from *Loxosceles* venoms [7]. We have shown that these toxins are the main components responsible for the cutaneous and systemic effects, including the induction of complement-dependent hemolysis, as well as the development of dermonecrotic lesions [7].

*Loxosceles* SMase D hydrolyzes sphingomyelin thereby generating ceramide-1-phosphate (C1P or N-acyl-sphingosine-1-phosphate) [8,9,10]. At low concentrations, C1P stimulates cell proliferation and inhibits apoptosis, whereas at relatively high concentrations it induces cell death by apoptosis [11]. Furthermore, *Loxosceles* SMase D can, in the presence of Mg^2+^, catalyze the release of choline from lysophosphatidylcholine (LPC), but not from phosphatidylcholine [12]. LPC is an abundant phospholipid in plasma, where it is tightly bound to albumin. Removal of its choline headgroup yields lysophosphatidic acid (LPA), a potent lipid mediator with numerous biological activities in many different cell types [13,14].

Our previous studies showed that development of the dermonecrotic lesion is associated with the induction and activation of matrix metalloproteinases (MMPs) [15,16]. In vitro and in vivo analysis showed that SMases D induced an increase in the expression of MMP-2 and MMP-9, decreased keratinocyte cell viability by apoptosis and increased the inflammatory infiltrate of neutrophils at the lesion site [15,16]. Moreover, the application of MMP inhibitors, such as tetracycline, doxycycline and minocycline, in in vitro and in vivo experimental models of cutaneous loxoscelism, inhibited the development of the skin lesion, concomitant with the inhibition of MMPs expression/secretion and keratinocyte apoptosis [16,17].

Various studies have shown the involvement of MMP-2 and MMP-9 in acute and chronic models of kidney injury, induced by ischemia-reperfusion injury, leading to glomerular damage, fibrosis and decline of renal function [18,19,20]. Tetracyclines have been used in the treatment of infectious diseases and are currently used in the treatment of diseases in which there is a participation of MMPs, such as osteoarthritis, ischemia, inflammation and cancer [21,22,23,24]. Tetracyclines inhibit MMPs by blocking the activity of the mature form, by chelation of zinc atoms from the active site, inhibition of maturation of pro-MMPs and reducing their expression [24,25].

The mechanism by which *Loxosceles* envenomation can cause kidney failure, one of the main pathologic aspects of systemic loxoscelism, is not fully understood. Using a murine model, we previously showed that animals injected with *Loxosceles* venom or SMase D, developed acute tubular necrosis with deposition of eosinophilic material in the proximal and distal tubules [26]. Edema, presence of erythrocytes in the extracellular space and vacuolar degeneration in the proximal and distal tubules, have also been described [27]. Some studies suggested that hemoglobinuria caused by *Loxosceles* envenomation can be an important component of the renal damage, by reducing circulation and leading to tubular necrosis [28]. Moreover, In vitro assays using the MDCK kidney cell line showed loss of cell viability upon treatment with SMase D, indicating direct toxicity of this toxin on kidney cells [29,30]. However, in vivo experiments using mice injected with *L. gaucho* venom demonstrated that kidney failure occurs due to renal vasoconstriction and intrarenal rhabdomyolysis and not directly by cytotoxicity [31].

Thus, considering that the mechanisms leading to renal failure in systemic loxoscelism is not fully elucidated and considering the important role of MMPs in the cutaneous loxoscelism as well as in various renal pathologies, the aim of this study was to investigate the role of extracellular MMPs in *Loxosceles* venom and SMase D induced renal cell damage. The potential of tetracycline in the control of venom induced renal pathology in vivo was also investigated.

## 2. Results

### 2.1. *Loxosceles* Venom and SMase D Induce Renal Cell Death by Apoptosis

**Figure 1:**
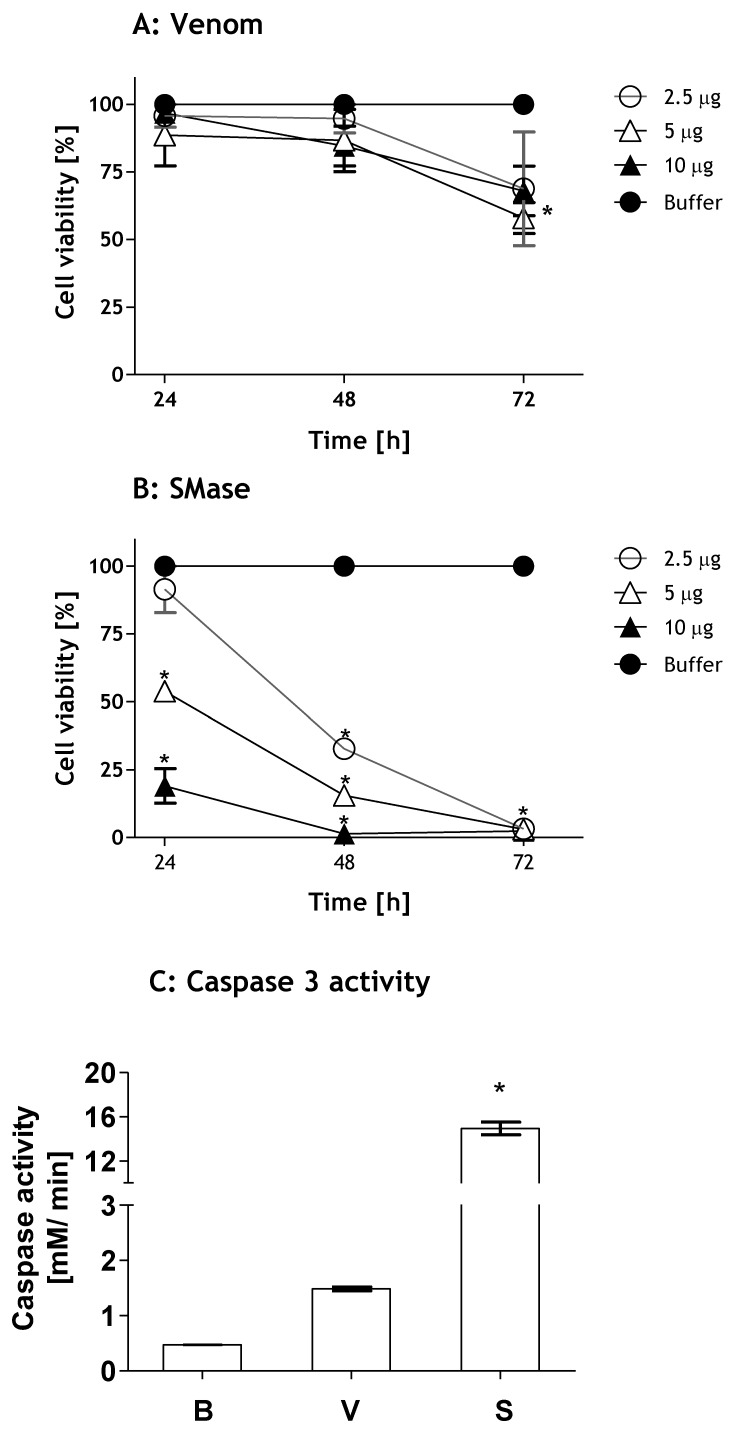
Toxicity of *Loxosceles* venom and SMase D on kidney cells. *Viability*: HK-2 cell cultures (5 × 10^4^ cells) were incubated with increasing concentrations of *Loxosceles* venom (V) (**A**) or SMase D (S) (**B**). After 24, 48 and 72 h of treatment, cell viability was analyzed by the MTT assay. *Caspase-3 activation*: HK-2 cells (10^6^) were incubated with 10 µg/ 200 µL total volume of venom or SMase D or Phosphate-buffered saline—PBS (B) for 3 h and the activity of Caspase-3 was analyzed in cell lysates (**C**). Results are representative of three independent experiments and expressed as the mean of duplicates ± standard deviation. Significant differences (*) *p* < 0.05 from the control (buffer treated cells).

To evaluate the toxicity of *Loxosceles* venom and SMase D, human HK-2 renal cells were incubated with venom or toxin and the cell viability was analyzed by the MTT method. Figure 1 shows that *Loxosceles* venom induced a significant loss of HK-2 cell viability after 72 h of treatment (Figure 1A). The SMase D was more potent than the venom, inducing cell death at 5 and 10 µg and at all times tested. After 48 h, 2.5 µg of SMase D also caused a significant decrease in cell viability. At 72 h of treatment, SMase D induced 100% cell death in all doses tested (Figure 1B).

In order to verify if the loss of cell viability induced by venom and SMase D occurred by apoptosis, HK-2 cells were incubated for 2 h with buffer, venom or SMase D (10 µg/10^6^ cells) and the supernatants analyzed for caspase-3 activity. Figure 1C shows that SMase D caused a significant caspase-3 activation, which was 10 times higher than that induced by whole venom, confirming that venom and SMase D both induced apoptosis.

### 2.2. MMP Secretion by HK-2 Cells

**Figure 2:**
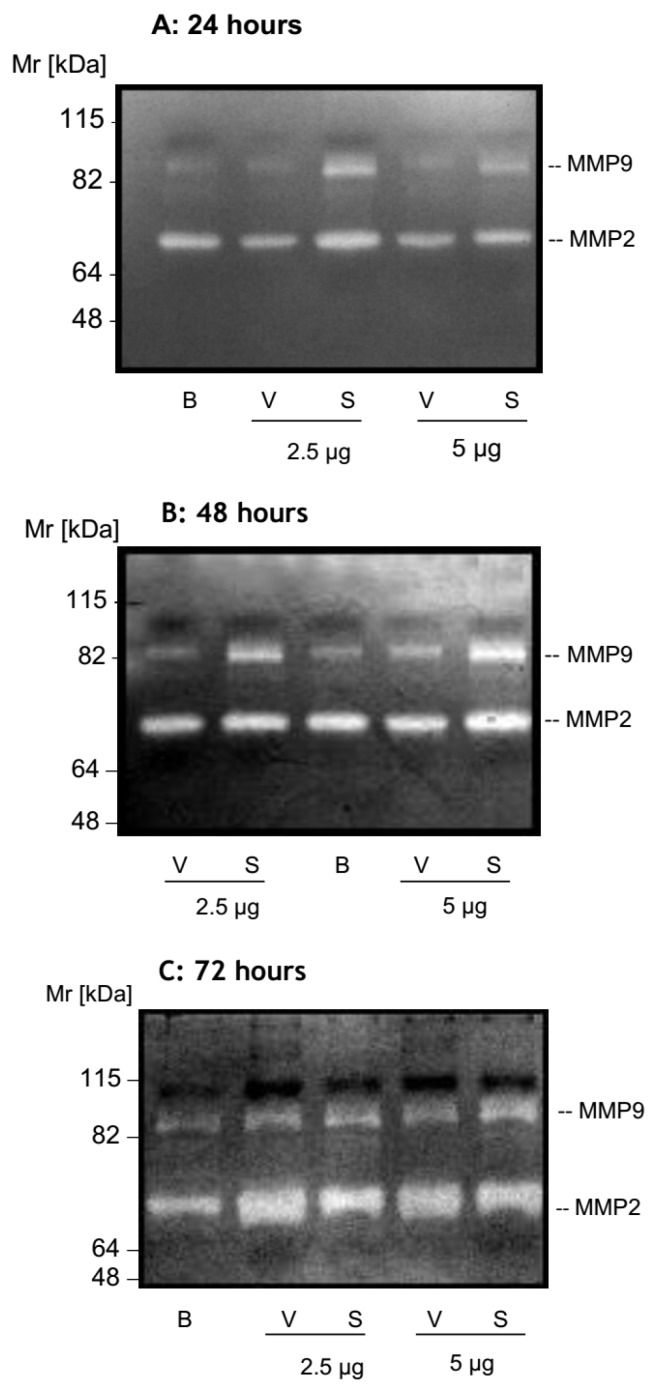
*Loxosceles* venom and SMase D induce the secretion of gelatinases in HK-2 cells. HK-2 cell culture supernatants collected after 24 (**A**), 48 (**B**) and 72 h (**C**) of treatment with *Loxosceles* venom (V) or SMase D (S) were run on gelatin containing 10% SDS-PAGE gels under non-reducing conditions. Control supernatants (B) were recovered at the same incubation periods from cells incubated with medium plus Phosphate-buffered saline—PBS (B).

To examine the involvement of metalloproteases in systemic loxoscelism, samples of supernatants of HK-2 cells treated with various concentrations of *Loxosceles* venom or SMase D or buffer were collected at 24, 48 and 72 h and subjected to zymography. Figure 2 shows that cells treated with 2.5 and 5 µg of SMase D induced an increase in the secretion of active MMP-9 protein (Mr ~85 kDa), at all time points when compared to untreated cells. Cells treated with venom showed higher secretion of MMP-9 after 48 and 72 h. Supernatants collected after 72 h of treatment with venom or SMase D, showed an increase in the secretion of a gelatinase with a Mr of around 70 kDa, corresponding to MMP-2 (Figure 2).

### 2.3. Tetracycline Prevents Venom and SMase D Increased Expression of MMPs and Protects Kidney Cells from Death

**Figure 3:**
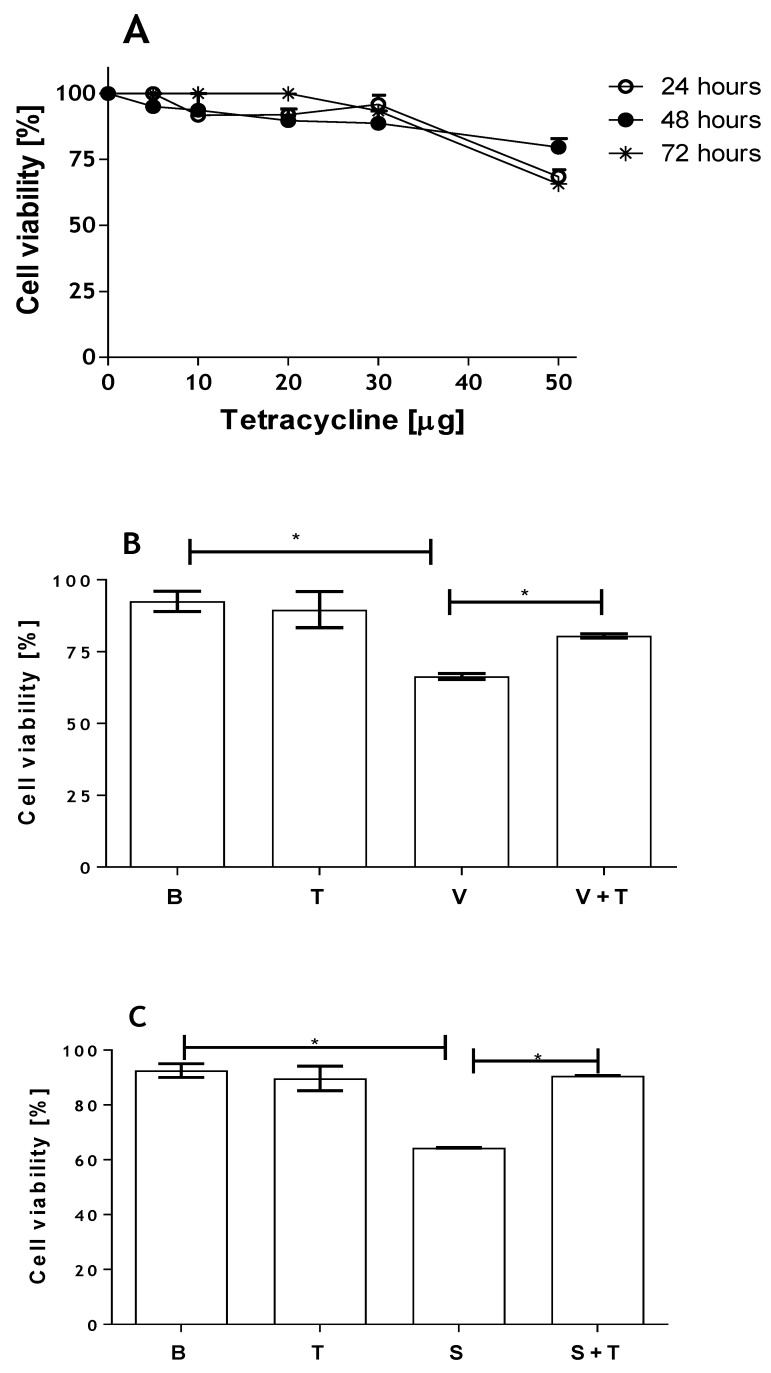
Tetracycline inhibits *Loxosceles* venom and SMase D induced cell death in HK-2 cells. HK-2 cell cultures (5 × 10^4^ cells) were incubated with increasing concentrations of tetracycline and cell viability was assessed at 24, 48 and 72 h by the MTT method (**A**); HK-2 cell cultures (5 × 10^4^ cells) were incubated with venom (V) (**B**) or SMase D (S) (**C**) from *L. intermedia* (5 µg/200 µL total volume), and treated or not with tetracycline (T) [20 µg/mL]. Analyses were performed after 48 h of incubation. All assays were performed in duplicate. Results are representative of three independent experiments and expressed as the mean of duplicates ± standard deviation. Significant differences (*) *p* < 0.05.

**Figure 4:**
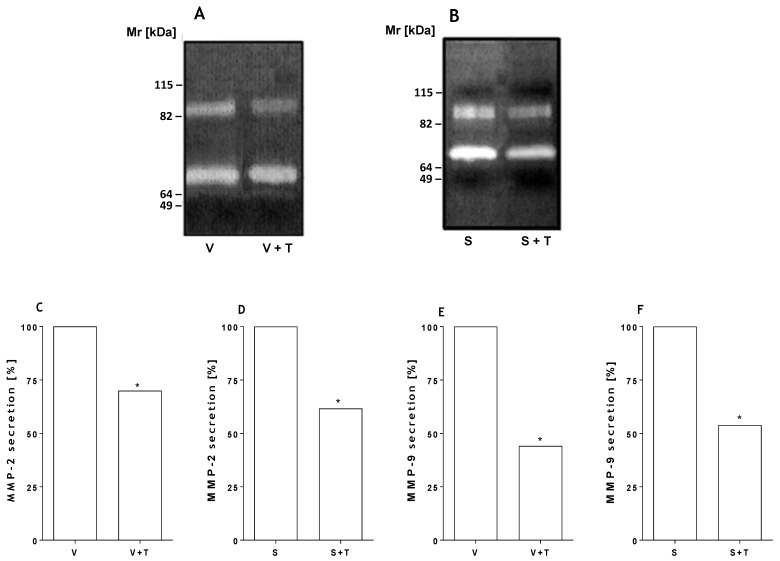
Tetracycline inhibits *Loxosceles* venom and SMase D induced MMPs. HK-2 cell cultures (5 × 10^4^ cells) were incubated with venom (V) (**B**) or SMase D (S) (**C**) from *L. intermedia* (5 µg/200 µL total volume), and treated or not with tetracycline (T) [20 µg/mL]. Supernatants of the HK-2 cells were collected after 48 h of incubation and submitted to zymography (**A**, **B**) followed by densitometry analyses (**C**–**F**). Significant differences (*) *p* < 0.05.

To assess whether tetracycline affected viability of human kidney cells, HK-2 cells were incubated with different concentrations of tetracycline and the viability was determined at 24, 48 and 72 h by the MTT method. Figure 3A shows that tetracycline at high concentrations (50 μg/mL) is toxic to kidney cells, causing loss of cell viability. At concentrations of 30 μg/mL tetracycline and lower, only a small reduction in cell viability was observed. In order to evaluate the possible protective effect of this inhibitor on cell death induced by the toxin and the venom, cells were incubated with SMase D or *Loxosceles* venom (5 μg) in the presence or absence of tetracycline (20 µg). Figure 3 (panels B and C) shows that the tetracycline treatment significantly prevented cell death induced by both venom and SMase D. This inhibition positively correlated with a significant decrease in the secretion of MMP-2 and MMP-9 as shown by zymography analysis (Figure 4).

### 2.4. *Loxosceles* Venom and SMase D Induce an Increase in MMP Gene Expression in HK-2 Cells

As shown in Figure 2 and Figure 4, both venom and SMase D from *Loxosceles* induced increased secretion of MMPs by HK-2 cells. To verify if this increase of MMPs protein activity in the HK-2 cells culture supernatants was due to increased expression rather than just increased secretion of these proteases, mRNA expression of the MMPs was assessed. Furthermore, the action of tetracycline on the expression of the gelatinases was also evaluated.

**Figure 5:**
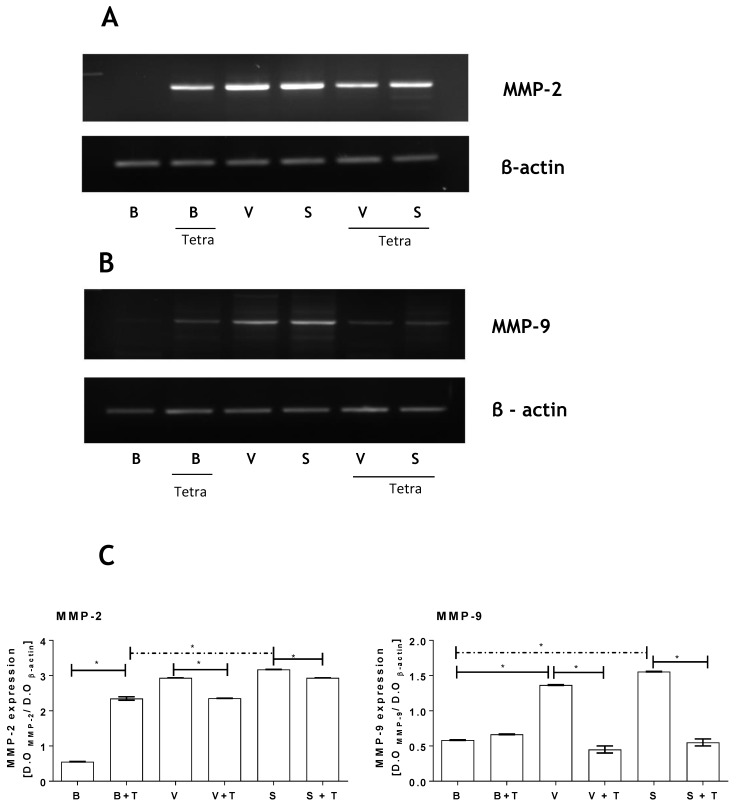
Tetracycline reduces MMP-2 and MMP-9 gene expression induced by *Loxosceles* venom and SMase D. HK-2 cell cultures (5 × 10^4^ cells) were incubated with venom (V: 12.5 µg/200 µL total volume) (**A**) or SMase D (S: 6.25 µg/200 µL total volume) (**B**) from *L. intermedia* and treated or not with tetracycline (T: 20 µg/mL). Cells treated with Phosphate-buffered saline—PBS (B) were used as negative control. MMP-2 and MMP-9 specific mRNA expression was analyzed at 48 h by RT-PCR. As control, mRNA expression of β-actin was assessed; (**C**) Densitometry analyses of MMP-2 and MMP-9 bands. Significant differences (*) *p* < 0.05.

Figure 5 (panels A and B) shows that venom and SMase D increased the mRNA expression of both MMP-2 and MMP-9 in HK-2 cells. Tetracycline on its own also increased the levels of mRNA expression of both MMPs, although not to the same level as the venom or SMase D. Co-incubation of the HK-2 cells with tetracycline and venom/SMase D prevented the venom/SMase D-induced increase in mRNA expression. The mRNA expression of β-actin was not affected by treatment with tetracycline, venom or SMase D. Figure 5C shows densitometric analysis of the MMPs expression; values were normalized with constitutive β actin expression.

### 2.5. Tetracycline Prevents *Loxosceles* Venom Induced Renal Injury in Mice

**Figure 6:**
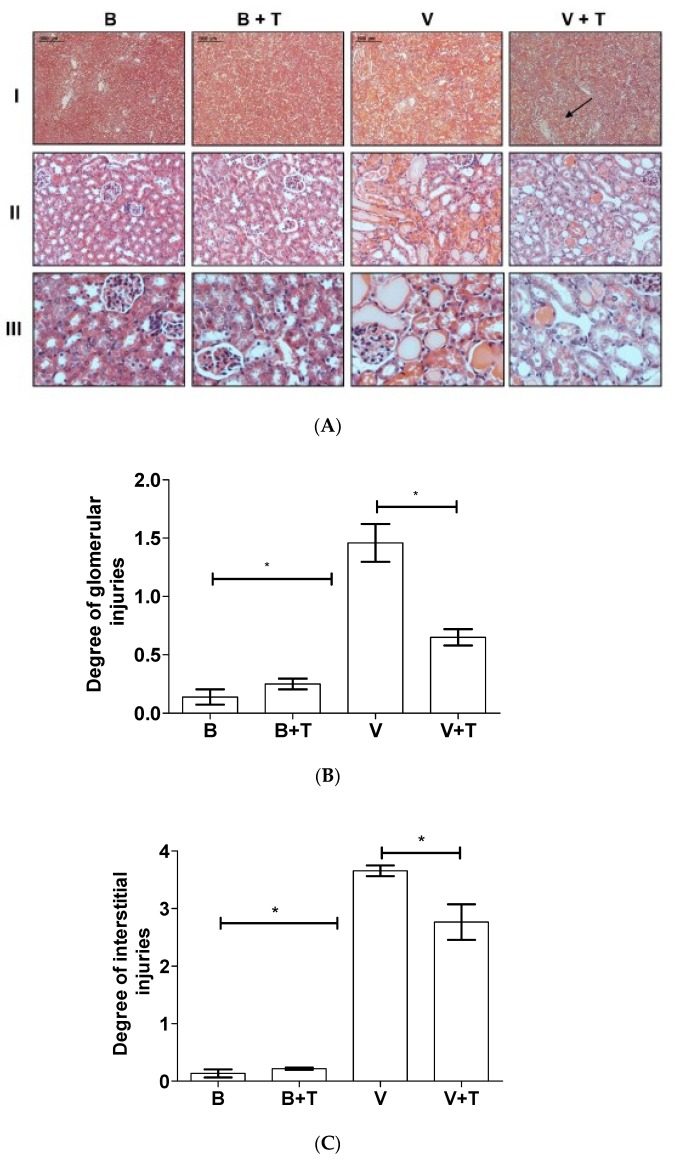
Histological analysis of the renal lesions induced by *Loxosceles* venom after tetracycline treatment. BALB/c mice were injected with 4.8 µg of venom (V) and, after 30 min, treated three times every 4 h with 200 µL of tetracycline (T) (5 mg/mL). Control animals were injected with Phosphate-buffered saline—PBS (B). After 24 h, the animals were euthanized and the kidneys collected for histological analysis. (**A**) Photos correspond to the sections of the kidney stained by H/E. Bars of 2000 μm, 1000 μm and 100 μm for lines I, II and III, respectively. Arrow shows the medullary region; Estimation of glomerular injuries (**B**) and interstitial changes (**C**) of the histological samples. Results are representative of two independent experiments and expressed as the mean of triplicates ± standard deviation. Significant differences (*) *p* < 0.05.

To evaluate the effect of tetracycline in renal injury in vivo, BALB/c mice were i.d. injected with *Loxosceles* venom. After 30 min, the animals were i.p. treated with tetracycline, which was repeated twice at intervals of 4 h. Control mice were injected with PBS or tetracycline only. After 24 h, the animals were euthanized and the kidneys collected for histological analysis. Figure 6 shows that kidneys obtained from control mice injected with PBS only and treated or not with tetracycline showed a normal pattern of renal tubules and renal corpuscles. Animals injected with venom showed dilation of the renal tubules, presence of hyaline material in the tubular and glomerular regions, and erythrocyte extravasation in the vascular region. Analysis of the tubular region showed significant interstitial injuries as loss of brush border and/or destruction of tubular structure. Changes could also be observed in the glomerular areas with fibrin exudation, thrombi formation, mesangiolysis and intra- or extracellular proliferation. The tetracycline treatment considerably reduced the renal injury induced by the venom, although some damage was still observed in the medullary region as indicated by the arrow (Figure 6).

**Figure 7:**
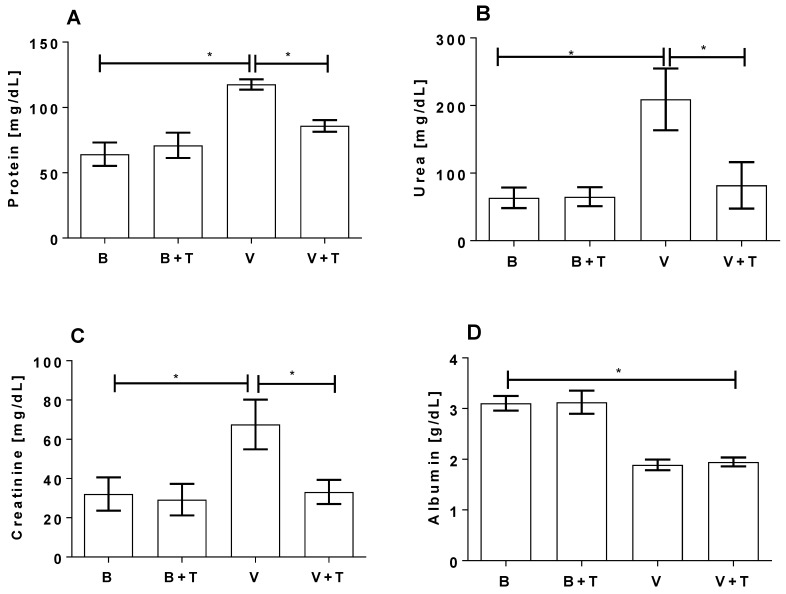
Biochemical analysis of serum and urine from *Loxosceles* venom and tetracycline treated group. BALB/c mice were injected with 9.6 µg of venom and, after 30 min, treated three times every 4 h with 200 µL of tetracycline [5 mg/mL]. Control animals were injected with Phosphate-buffered saline—PBS (B). Urine samples, collected from each animal during 24 h of the treatments, were analyzed for the presence of protein (**A**) and creatinine (**B**). Serum samples, collected 24 h after buffer/venom injection, were analyzed for the presence of urea (**C**) and albumin (**D**). Results are representative of two independent experiments and expressed as the mean of triplicates ± standard deviation. Significant differences (*) *p* < 0.05.

Serum and urine samples from the same mice were collected for biochemical analysis. As shown in Figure 7 venom induced a significant increase of creatinine and protein in the urine and a significant increase of urea and a significant decrease of albumin in the serum samples, as compared to control animals injected with buffer or buffer plus tetracycline. The treatment of mice with tetracycline reduced the venom-induced changes of creatinine and protein in the urine and of urea in the serum, but not of albumin in the serum.

## 3. Discussion

Envenomation by spiders of the *Loxosceles* genus can cause severe cases kidney failure and death. It is well accepted that SMase D is the main venom component responsible for most of the clinical aspects observed in loxoscelism. We have previously shown that both the venom and SMase D of *Loxosceles* are capable of inducing loss of viability of human keratinocytes [16].

Similar to the data obtained in studies using canine kidney cells (MDCK) [32], in the present study we demonstrate that *L. intermedia* venom is toxic to human kidney cells (HK-2), causing 30%–40% cell death after 72 h of treatment. The SMase D was more toxic, causing 100% cell death with only 5 µg of protein after 48 h. After 72 h incubation, 100% cell death at all concentrations of SMase D was observed, indicating that the toxic action of SMase D may contribute to the development of the renal damage.

The matrix metalloproteases (MMPs) belong to a family of zinc-dependent endopeptidases secreted or bound to the cell membrane, which can be classified into several subgroups, such as collagenase, elastase, gelatinase, and matrilysins metalloproteases. They are capable of hydrolyzing a variety of proteins, including, extracellular matrix components such as collagen and fibronectin and cell surface molecules [33,34,35]. Increase of MMPs in inflammatory renal disease leads to the degradation of the extracellular matrix [36]. MMPs are also reported to be associated with other nephropathies such as proteinuria, loss of membrane selectivity, increased glomerular fibrosis and renal epithelium [27,37,38].

In previous studies, we have demonstrated in an in vivo rabbit model of dermonecrosis and in In vitro using human keratinocytes (HaCaT cells), that venom and SMase D from *L. intermedia* induced increased secretion of MMPs (MMP-2 and MMP-9) and that this directly correlated with the loss of cell viability by apoptosis [15,16,17]. The results obtained in the present study showed that both venom and SMase D are also able to induce an increase in the expression and secretion of endogenous metalloproteinases MMP-2 and MMP-9, as well as activation of caspase-3 in the human proximal tubular cell line HK-2. Apoptosis is an important mechanism leading to cell death, which is initially characterized by a series of morphological changes, DNA fragmentation and exposure of phosphatidylserine on the cell surface [39]. The activation of caspase-3, a protein belonging to the family of cysteine proteases that cleaves substrates with aspartate residues, plays a central role in the programmed cell death [40]. The results obtained here suggest that the HK-2 cell death induced by venom/SMase D from *Loxosceles* occurs by an apoptotic process.

Our previous In vitro studies, using human keratinocytes, showed that tetracycline inhibits the toxic action of the venom and SMases D on cell viability, and also prevents the venom and SMase D increased secretion of MMP-2 and MMP-9 [16]. Using rabbits as model of cutaneous loxoscelism we showed that the topical use of tetracycline significantly reduced the dermonecrotic lesion induced by *Loxosceles* venom or SMase D. This result correlated with a decreased expression/secretion of MMPs at the injection site of toxins [17]. In the present study using the renal cell line HK-2, we show that tetracycline significantly reduced the venom and SMase D-induced HK-2 cell death and inhibited the venom/SMase D increased expression and secretion of MMPs, as demonstrated by PCR and zymography analyses. Although tetracycline itself seems to induce some toxicity, this was compensated for by its ability to completely prevent the much higher toxicity induced by the venom or SMase D.

Interestingly, it was recently demonstrated that the bioactive sphingolipid ceramide 1-phosphate (C1P), one of the products of SMase D catalytic activity on cell membrane sphingomyelin [8,9,10], is able to upregulate MMP-2 and MMP-9 in J774A.1 macrophages [41]. Ceramide and its phosphorylated counterpart are recognized as “bioactive sphingolipids” and they participate in signal transduction pathways, regulating various different cell functions such as proliferation, differentiation, adhesion and cell death [42]. The role of CIP in the increased expression/secretion of MMPs and in cell death in cutaneous and systemic loxoscelism will be further investigated.

We showed here that tetracycline significantly reduced renal injury in animals injected with the venom after 24 h of treatment, as demonstrated by a reduction in the histopathology and in the secretion markers of renal injury in urine and serum. The tetracycline group of antibiotics is increasingly being used for treatment of non-infectious diseases because of its effect on metalloprotease synthesis and activity. Several studies have demonstrated the role of MMPs in renal injury, including that caused by ischemia reperfusion (I/R) [18,19,20,36,37]. In addition, several studies demonstrated that doxycycline and minocycline inhibited MMP-2 and MMP-9 expression in animal models of ischemia reperfusion injury and thereby reduced the pathology. In rats subjected to renal I/R, increased MMP-2 expression was reduced by tetracycline, which also reduced apoptosis [19,20,36]. Our observation that tetracycline can inhibit the *Loxosceles* venom induced renal pathology, suggests that tetracyclines could be used in the treatment of renal pathology in systemic loxoscelism.

Serum and urine were collected from animals injected with venom and/or tetracycline for evaluation of the presence of protein, albumin, creatinine and urea, as indication of renal injury [43]. *Loxosceles* venom induced a significant increase in creatinine and protein secretion into the urine and generation of urea in the serum, which was largely prevented by the administration of tetracycline. However, while the venom also induced a significant decrease in serum albumin levels this was not prevented by tetracycline. Reduced levels of albumin can be present in acute renal syndrome. This can be related to the inflammatory process, in which reduction of the albumin concentration in the circulation occurs due to a decrease of osmotic pressure by an increase of the reabsorption of sodium and water [44].

## 4. Conclusions

The data presented here, using In vitro and in vivo models, suggest that activation of MMP-2 and MMP-9 is involved in renal injury induced by *Loxosceles* venom and its main toxin SMases D and that the use of tetracycline may have a protective effect on *Loxosceles* venom and SMase D induced kidney damage and could potentially aid the treatment of systemic loxoscelism.

## 5. Material and Methods

### 5.1. Chemicals, Reagents, and Buffers

Tween 20, bovine serum albumin (BSA), formalin, gelatin, Triton X-100, 3-(4,5 dimethylthiazol-2yl)-2,5 diphenyltetrazolium bromide-MTT and tetracycline were from Sigma (St. Louis, MO, USA). Brij-35 was from Fluka—BioChemika (Werdenberg, Switzerland). Coomassie brilliant blue solution: 40% methanol, 10% acetic acid and 0.1% Coomassie brilliant blue. Buffers were: Phosphate-Buffered Saline (PBS), pH 7.2, containing 10 mM NaH_2_PO_4_, 150 mM NaCl; Zymography buffer, pH 8.3: 50 mM Tris-HCl, 200 mM NaCl, 10 mM CaCl_2_, 0.05% Brij-35.

### 5.2. Venom

*Loxosceles intermedia* spiders were provided by “Laboratório de Imunoquímica, Instituto Butantan, SP, Brazil”. The venom was obtained by electrostimulation by the method of Bucherl [45], with slight modifications. Briefly, 15–20 V electrical stimuli were repeatedly applied to the spider sternum and the venom drops were collected with a micropipette, aliquoted and stored at −20 °C. The permission to access the *Loxosceles* venom (permission No. 01/2009) was provided by the Brazilian Institute of Environment and Renewable Natural Resources (IBAMA), a Brazilian Ministry of the Environment’s enforcement agency.

### 5.3. Sphingomyelinase D Expression

Recombinant *L. intermedia* SMase D named as P1 (accession number: AY304471.2) was expressed in *Escherichia coli* strain BL21 (DE3), as a fusion protein composed of the mature SMase D with a N-terminal extension containing a 6× histidine tag, and purified as described [46]. The protein content of the samples was evaluated by the Lowry method [47].

### 5.4. Cell Culture and Maintenance

The human kidney cell line HK-2 [48] was maintained in 50% DMEM and 50% RPMI-1640 medium (Gibco-BRL, Gaithersburg, MD, USA), supplemented with 10% fetal bovine serum (FBS), 100 IU/mL penicillin and streptomycin at 37 °C in humidified air with 5% CO_2_.

### 5.5. Cell Viability

Cell viability was analyzed by the MTT [49] assay with slight modifications. Briefly, HK-2 cells were sub-cultured in 96-well plates in fetal bovine serum (FBS) containing culture medium at 5 × 10^4^ cells/200 µL. After 24 h, the cells were maintained overnight in culture medium without FBS and then incubated with different concentrations of *Loxosceles* venom or SMase D in the presence or absence of increasing concentrations of tetracycline. Cells incubated with medium plus PBS were used as positive control (100% viable). After the indicated periods of incubation (24, 48, 72 h), the culture supernatants were removed and the cells were incubated with 60 µL/well of MTT solution in medium and the plates incubated for 30 min at 37 °C/5% CO_2_. Supernatants were removed and replaced with 100 µL of DMSO. The absorbance of the samples was measured in a spectrophotometer (Multiskan-EX, Labsystems, Finland) at 540 and 620 nm. The relative cell viability was calculated as: [(sample OD_(540–620 nm)_ − background control OD_(540–620 nm)_/(control OD_(540–620 nm)_ − background OD_(540–620 nm)_] × 100.

### 5.6. Caspase-3 Activity Assay

Apoptosis was assessed using the Caspase-3 kit according to the manufacturer’s recommendations (Roche Molecular Biochemicals, Pleasanton, CA, USA). Briefly, HK-2 cells treated with venom or SMase D (10 µg/10^6^ cells), for 2 h at 37 °C, were lysed with the lysis buffer and centrifuged for 1 min, 20 °C at 14,000 rpm. The supernatant was removed and stored at −20 °C until use. 96-well plates were sensitized with anti-caspase-3 monoclonal antibody and incubated at 4 °C overnight. Blocking solution was added to all wells and the plates were incubated for 30 min at room temperature. The plates were washed with wash solution and 100 μL of each experimental sample were added and the plates were incubated for 1 h at 37 °C. The plates were washed, substrate solution (Ac-DEVD-AFC) was added to the wells and the plates were incubated at 37 °C. Activation of Caspase-3 was determined after 3 h using a VICTOR3™ spectrofluorimeter (Perkin-Elmer, Waltham, MA, USA), using excitation and emission wavelengths of 405 nm 535 nm, respectively. The Caspase-3 activity was expressed as the amount of Caspase-3-induced release of the fluorescent AFC (7-amido-4-trifluoromethyl coumarin) *per* minute.

### 5.7. Gelatin Zymography

Supernatants from HK-2 cells incubated with venom or SMase D, in the presence or absence of tetracycline, were mixed with non-reducing sample buffer and run on SDS-PAGE (10% polyacrylamide gel containing 1% gelatin) [50]. Gels were washed twice for 30 min at room temperature in 2.5% Triton X-100, and incubated overnight at 37 °C in zymography buffer. Gels were stained in Coomassie Brilliant Blue solution for analysis of the presence of clear zones, which indicates proteolytic digestion [51].

### 5.8. RNA Extraction

HK-2 cells were sub-cultured in 24-well plates in DMEM/RPMI medium plus FBS at 5 × 10^5^ cell/mL at 24 h. Subsequently, the cultures were maintained overnight in medium without FBS followed by incubation with venom (12.5 µg) or SMase D (6.25 µg) in the presence or absence of tetracycline at 37 °C/5% CO_2_. After 48 h, total RNA was extracted from the cells using RNAspin Mini RNA Isolation kit (GE Healthcare, Pittsburgh, PA, USA). The RNA was aliquoted and stored at −80 °C until use.

### 5.9. RT-PCR and PCR

RNA samples were subjected to reverse transcription reaction using the Super Script Plasmid System for cDNA Synthesis (Invitrogen Carlsbad, CA, USA) to obtain the cDNA, according to manufacturer’s instructions. The transcribed cDNA was then amplified by PCR using the following primers for MMP-2 (*sense*: 5′-CAC CTA TAC CAA GAA CTT CCG-3′, *anti-sense*: 5′-CAG GAG GAG AAG GCT GTG TT-3′), MMP-9 (*sense*: 5′-TGG ACG ATG CCT GCA ACG TG-3′, *anti-sense*: 5′-GTC GTG CGT GTC CAA AGG CA-3′) and β-actin (*sense*: 5′-CCT TCC TGG GCA TGG AGTC-3′, *anti-sense*: 5′-GAG GAG CAA TGA TCT TGA TCT TC-3′). PCR products were analyzed on 2% agarose gel and specific bands were quantified by densitometry (Gel Logic 100, Kodak, Rochester, NY, USA).

### 5.10. Mice

BALB/c male mice, aged 2 months and weighing 18–22 g, were obtained from the Central Animal Breeding unit at the Butantan Institute, SP, Brazil. The animals had *ad libitum* access to water and food. All experimental procedures involving animals were in accordance with the ethical principles in animal research adopted by the Brazilian Society of Animal Science and the National Brazilian Legislation no.11.794/08. The protocol was approved by the Institutional Committee for the Care and Use of Laboratory Animals from Butantan Institute (CEUAIB 698-10).

### 5.11. Treatment of Mice with Loxosceles Venom and Tetracycline

Mice (6 per group) were kept throughout the experiments in individual metabolic cages. Samples of 200 µL of *L. intermedia* venom (48 µg/mL) in PBS were injected intradermally (i.d.) in the dorsal region of mice. Thirty minutes later, the animals were injected with 200 µL of tetracycline (5 mg/mL) intraperitoneally (i.p.), which was repeated twice at 4 hour intervals. Negative control groups were animals injected with PBS and treated as the experimental groups. After 24 h, the mice were euthanized and the kidneys collected for histological analysis; serum and urine were also collected for biochemical analysis.

### 5.12. Biochemical Analysis

Urine and serum samples were collected from control and envenomed animals, treated or not with tetracycline, and analyzed for the presence of urea and albumin in serum samples collected after 24 h, and creatinine and protein in urine samples collected during 24 h, using specific kits (Labtest Diagnóstica SA, Minas Gerais, Brazil).

### 5.13. Histological Analysis

Twenty four hours after injection, the kidneys of animals were collected and fixed in 10% buffered formalin solution and embedded in paraffin. Tissue sections were stained with hematoxylin and eosin and examined for the presence of bleeding and tissue damage. Analysis of the degree of interstitial changes were estimated as 0: minimum, 1: tubular injuries were <25%, 2: 25%-50%, 3: 50%-75%, 4: 75%; degree of pathological changes in the glomeruli were estimated: 0: minimum, 1: <10%, 2: 10%-30%, 3: 30%-60%, 4: 60%

### 5.14. Statistical Analysis

The results were expressed as the mean ± SD. Data were analyzed statistically by the ANOVA and Tukey test in the in vitro assays. *p*-values ≤ 0.05, 0.1 and 0.001 were considered significant. In vivo assay data were analyzed statistically by the Kruskal-Wallis and F test. Statistical analyses were performed using GraphPad Prism software (version 7, La Jolla, CA, USA).
